# Numerical learning of deep features from drug-exposed cell images to calculate IC50 without staining

**DOI:** 10.1038/s41598-022-10643-9

**Published:** 2022-04-22

**Authors:** Kookrae Cho, Eun-Sook Choi, Jung-Hee Kim, Jong-Wuk Son, Eunjoo Kim

**Affiliations:** Division of Electronics and Information System Research, Daegu Gyeongbuk Institute of Science and Technology (DGIST), Techno-Jungangdaero 333, Daegu, 42988 Republic of Korea

**Keywords:** Imaging, Software

## Abstract

To facilitate rapid determination of cellular viability caused by the inhibitory effect of drugs, numerical deep learning algorithms was used for unlabeled cell culture images captured by a light microscope as input. In this study, A549, HEK293, and NCI-H1975 cells were cultured, each of which have different molecular shapes and levels of drug responsiveness to doxorubicin (DOX). The microscopic images of these cells following exposure to various concentrations of DOX were trained with the measured value of cell viability using a colorimetric cell proliferation assay. Convolutional neural network (CNN) models for the study cells were constructed using augmented image data; the predicted cell viability using CNN models was compared to the cell viability measured by colorimetric assay. The linear relationship coefficient (r^2^) between measured and predicted cell viability was determined as 0.94–0.95 for the three cell types. In addition, the measured and predicted IC50 values were not statistically different. When drug responsiveness was estimated using allogenic models that were trained with a different cell type, the correlation coefficient decreased to 0.004085–0.8643. Our models could be applied to label-free cells to conduct rapid and large-scale tests while minimizing cost and labor, such as high-throughput screening for drug responsiveness.

## Introduction

In vitro high-throughput assays for screening drug responsiveness are increasingly needed not only for medical and pharmaceutical research, but also for clinical purposes using cell lines, patient-derived primary cells, or organoids. In this context, automatic analysis of drug responsiveness using cell images have facilitated advanced microscopic technology such as counting cell numbers and discriminating between live and dead cells to determine in vitro responses^1,2^.

Recently, artificial intelligence-involved instrumental analysis has improved the experimental process by using accumulated data from researchers as training data to predict analytical results, reducing time, cost, and labor. Deep-learning-based training has been applied to obtain optimized results for the counting of cells with fluorescence or colorimetric staining^3,4^. An image-based learning approach was also applied to the discrimination of live and dead cells following the fluorescence staining of cells after drug treatment^5,6^. The continuous values of labels assigned to cell images were predicted by performing numerical deep learning on label-free input images using a recent image analysis technique^7,8^. In these studies, the continuous values of fluorescent intensity from stained cells were assigned to non-stained light microscope images, and then the fluorescent intensity was predicted using the label-free cell images.

To provide numerical values of cell viability without staining, label-free cell images could be assigned with measured values obtained by colorimetric cell proliferation assay and used as input data for numerical learning. In this case, it would be possible to predict 50% inhibitory concentration (IC50) based on precisely predicted cell viability from the training of cell images. Using this model, high-throughput drug responsiveness screening could be achieved by rapid and automatic analysis saving time, cost, and labor.

In this study, we provide numerical deep learning results using label-free cell images, which were used to determine IC50 values based on the predicted cell viability in cell culture dishes. The ground truth label was the cell viability indicator, optical density at 450 nm (OD_450 nm_), which was obtained by cell proliferation assay following drug treatment of cells. Cell viability can be defined as the number of cells that are either alive or dead after undergoing drug treatment. The prediction result was compared to the measured values, and the correlation coefficient and statistical difference between the measured and predicted data were evaluated. The drug doxorubicin (DOX) was used to induce cytotoxicity in three types of cell lines: A549, HEK293, and NCI-H1975. These cell lines were used to prepare label-free cell images.

Because an efficient method for cell viability prediction is critical for various cellular culture-based experiments, including anti-cancer drug discovery, a web-based algorithm for image-based IC50 determination without staining of cells was introduced to support high-throughput image analysis with low cost and time requirements. Finally, the prediction result of cell viability and IC50 values was evaluated in aspect of possible alternative method to replace the conventional colorimetric cell proliferation assays.

## Materials and methods

### Cell lines and cultures

Human epithelial lung carcinoma A549 cells (CCL-185), human kidney HEK293 cells (CRL-1573), and human non-small cell lung cancer NCI-H1975 cells (CRL-5908) were purchased from the American Type Culture Collection (ATCC, Manassas, VA, USA). The cells were cultured in Gibco RPMI-1640 culture medium (Thermo Fisher, Waltham, MA, USA) supplemented with 10% fetal bovine serum albumin (FBS, Thermo Fisher, Waltham, MA, USA). During the culture process, the cells were placed in a humidified incubator at 37 °C with 5% CO_2_.

### Measurement of cell viability

To prepare images of the cultured cells corresponding to the molar concentration of a drug to inhibit normal cellular growth, DOX was added to A549, HEK293, and NCI-H1975 cells in concentrations ranging from 0.001 nM to 100 μM (total 11 concentrations). For each DOX concentration, eight repetitive samples were prepared, and three sets of experiments were performed separately for the A549, HEK293, and NCI-H1975 cell types. Briefly, cells were seeded into 96-well plates at a density of 1 × 10^3^ cells/well, and DOX was added to each well at a selected concentration after 24 h of incubation. After 48 h of incubation, the cell images from each well were captured by light microscopy (Leica DMI3000B, Leica Microsystems, Wetzlar, Germany) equipped with a Zyla sCMOS camera (Andor Technology Ltd, Belfast, UK). An image for each well was captured, and every sample was in the center of the field of view.

Cell viability was then determined using Cell Counting Kit-8 (CCK kit, Dojindo Molecular Technology Inc., Rockville, MD, USA), according to the manufacturer’s instructions. The OD_450 nm_ for the CCK assay was measured using a spectrophotometric microplate reader (Promega, Madison, WI, USA).

### Image pre-processing

We used bright-field images of the cells in 96-well plates as input variables. For the classification of learning data, each cell image was assigned quantitative labels of cell viability data (OD_450 nm_) following 48 h of DOX exposure, which was also taken as the expected output. Before predicting drug responsiveness using label-free cell images and further calculation of IC50, we performed an exploratory analysis on the actual dataset. For all the dataset images, we constructed 1028-dimensional feature vectors using MobilenetV2, which was pre-trained using the ImageNet dataset (https://www.image-net.org/) without classification layers. Patterns such as clustering, abnormality, and outliers were obtained as the training results from the learned feature vectors.

In addition, we performed data augmentation for the training images, artificially increasing the diversity and size of the training samples to reduce the required number of datasets^9,10^. This included using random 90-degree rotations, random cropping (to 70% of the original size), and vertical/horizontal flipping of each image. All the original images are in RGB, JPG format with a resolution of 2160 × 2560 pixels. The training images were cropped to 1512 × 1792 pixels which is 70% of the original image. In addition, the images were adjusted for brightness in the range [− 0.2, 0.2], saturation in the range [0.6, 1.6], contrast in the range [0.7, 1.3], and hue in the range [–0.08, 0.08].

### Model construction for the prediction of cell viability

For model training, transfer learning was applied using MobileNetV2 architecture and feature weights, which were pre-trained for the ImageNet dataset as described earlier. The MobileNetV2 architecture without the top classification layer was used as the base model for the CNN image analysis. One fully connected layer with 10 hidden nodes was linked to it, followed by another fully connected layer that outputs the prediction result. The frozen base model was used as a feature extractor, and only the added layers were trained using the base model.

Among the 264 images labeled with OD_450 nm_ values determined by the cell proliferation assay, 198 images were used for training and validation and 66 images were reserved for testing. Original (unaugmented) images were used to predict cell viability, with the splitting ratio of the dataset being 6:4. For a regression analysis, the numerical output for each cell image was predicted as a drug responsiveness score and compared it to the measured values determined by the cell proliferation assay. To support the test results, we additionally performed fourfold cross validation. Each fold had 25% of the data set without repetition. In addition to MobileNetV2, Inception V3^11^ and InceptionResNet V2^12^ models were also trained and applied for fourfold cross validation.

### Visualization of feature attributions

We applied two visualization techniques to investigate the behavior of the trained model. First, we extracted the feature vectors of the test images from the output of the trained model without the final prediction layer. These high-dimensional feature vectors were transformed to low-dimensional features using two-dimensional (2D) t-distributed stochastic neighbor embedding (t-SNE)^13^. Next, we adopted Google Explainable AI with XRAI option (https://cloud.google.com/explainable-ai)^14^ to display the attributes of features in the CNN model.

### Determination of IC50 by doxorubicin

Based on the changes in cell viability following treatment of cells with DOX, IC50 values were determined from non-linear regression curves fitted to DOX concentration and OD_450 nm_ values, according to the following Hill Eq. (1), where Max and Min are the maximum and minimum values of OD_450 nm_ in the sigmoidal regression, respectively.

1 $$Y = Min + \frac{Max - Min}{{1 + \left( \frac{X}{IC50} \right)^{Hill\,coefficient} }}.$$

The measured IC50 was calculated using the least square fit of four parameter-sigmoidal curves executed using Prism ver. 9 (GraphPad Software, San Diego, CA, USA).

### Web application

We developed a web application that allows a user to predict drug responses from cell images and calculate the IC50 from the predicted responses. First, we used the Python-based TensorFlow to build and train the model according to the structure and method mentioned earlier. Then, the trained model was saved in HDF5 standard format and converted to TensorFlow.js Layers format for use in the browser (Fig. S1). The converted model files were loaded from a URL where the hosted model files were hosted. When a user uploads the images of the cells that had been treated with a specific concentration of drugs to a web browser, the drug responsiveness in cell viability can be predicted, allowing for the IC50 to be calculated at once. Three types of models were developed separately, predicting drug responsiveness by calculating the IC50 based on the A549, HEK293, and NCI-H1975 cell images.

### Statistical analysis

Comparison between the measured and predicted values was performed using the correlation coefficient r^2^. Unpaired and paired *t* tests were also performed for groups of measured and predicted values using Prism ver. 9.3.0 (GraphPad Software, San Diego, CA, USA, https://www.graphpad.com/scientific-software/prism/). Differences between measured and predicted values were calculated as average (AVE) ± standard deviation (SD) from the paired *t* test.

## Results and discussion

### Images obtained for training of the deep learning model

**Figure 1:**
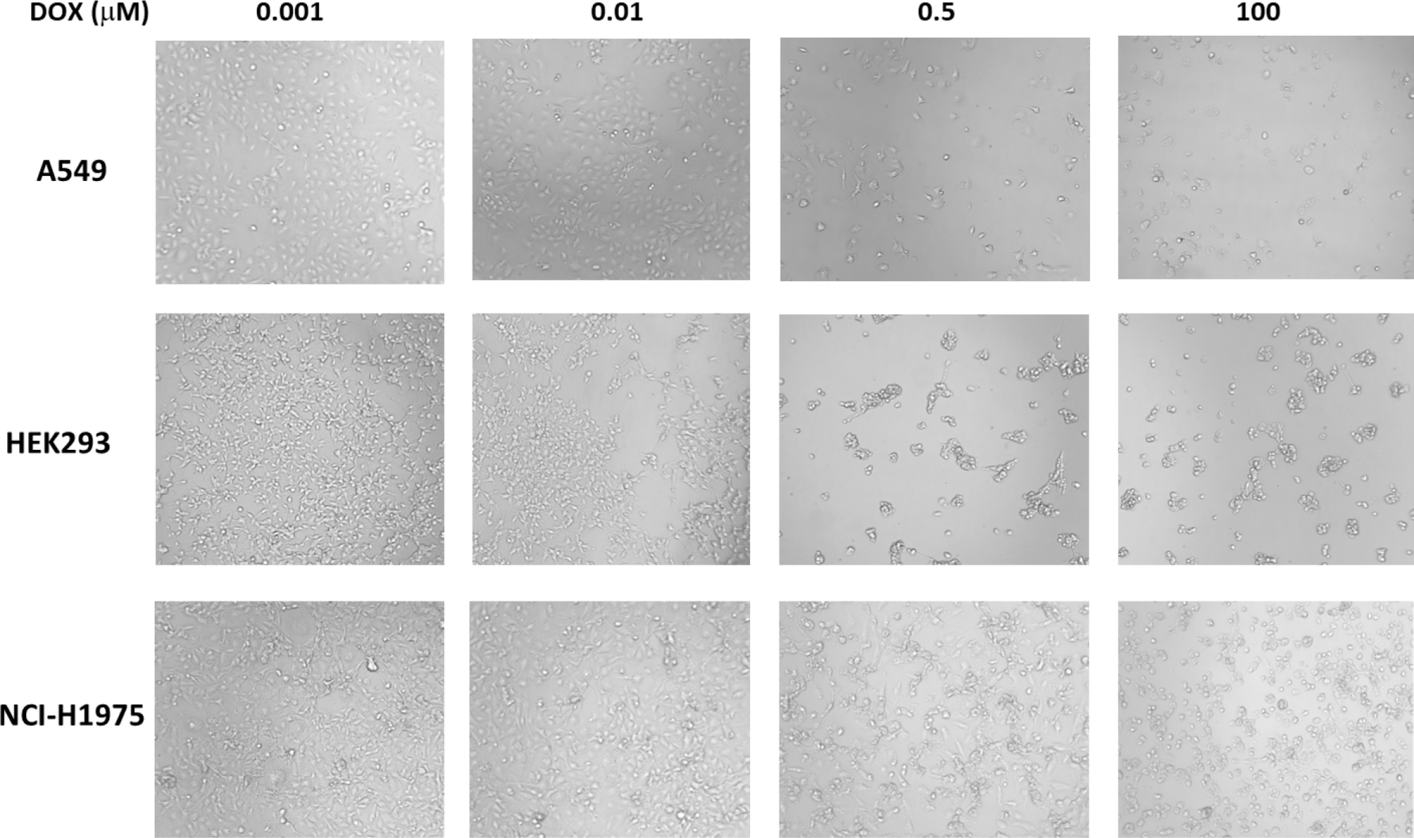
Representative cell images used in this study. The images were captured by light microscope with a magnification ratio × 100.

Three cell types, A549, HEK293, and NCI-H1975, were treated with DOX at concentrations of 0.0001, 0.001, 0.005, 0.01, 0.05, 0.1, 0.5, 1, 5, 10, and 100 μM in 96-well cell culture plates. For each of the 11 concentrations, eight repetitive samples were prepared, and three sets of 11 concentration × 8 repetitive samples were prepared for the numerical learning of bright-field cell images. The images of cells in 96-well plate wells under the given concentration of DOX were captured using a light microscope at × 100 magnification and used as input data for image classification to predict the cell viability and IC50 quantitatively. Representative cell images are shown in Fig. 1.

### Convolution of neural network model construction to predict drug responsiveness

In this study, we propose a CNN-based regressor that predicts the drug responsiveness of cells from their bright-field images. The CNN model was constructed using MobileNet2, which is very similar to the original MobileNet, except that it uses inverted residual blocks with bottlenecking features for analysis^15^. MobileNet is suitable for mobile vision applications because it dramatically reduces network complexity and model size^16^. The model was trained using unlabeled cell images with the assigned cell viability scores as inputs. These cell viability scores were determined by a cell proliferation assay using the colorimetric method, with continuous values ranging between 0 and 2. By setting the cell viability predictions as expected outputs, the responsiveness to DOX was estimated using the IC50 indicator for three types of cells: A549, HEK293, and NCI-H1975.

In this study, data augmentation and transfer learning were performed to induce high performance in the accuracy of the prediction, showing that even a relatively small dataset (264 images for each cell type) could be used in numerical learning. Data augmentation techniques can be used to overcome the limited number of available data by artificially increasing the diversity and size of the training samples^9,10^. In this study, training images were augmented by performing operations such as zoom, flip, and rotate. When data augmentation such as random crop or zoom operation were applied to train a model that predicts continuous values, the augmented images might not reflect the absolute response. Therefore, the size of the cropping or zooming of the original image should be predetermined to sufficiently reflect the cell distribution of the original cell images. In this study, the responsiveness of drugs represented by IC50 was determined by the relative 50% inhibition concentration between 0 and 100% inhibition, rather than being determined by the absolute number of dead or living cells. Therefore, it was required to determine the appropriate random crop size to accurately predict the IC50 value while increasing the amount of training data by data augmentation. We randomly cropped the original image to 70% size, computed the feature vectors, and performed 2D t-SNE visualization. Figure S2 presents the 2D t-SNE visualization of the augmented image embedding. Images augmented at the same concentration formed a cluster, with a distance maintained between different clusters. It was expected that a random crop of 70% size would be optimal for data augmentation in this study. As a result, the data augmentation process is suitable for pre-processing the model construction.

Among 264 cell proliferation images for each cell type, 198 images were used for training and validation, and 66 images were kept for testing. The splitting ratio of the dataset used in this analysis (6:4) was different from the commonly suggested ratio for training, validation, and testing (8:1:1)^17^. This was because a set of testing images were composed of eleven images corresponding to the eleven concentration of DOX: six sets of experimental results were used as testing sets.

Finally, three types of models, A549-M, HEK293-M, and NCI-H1975-M, were constructed, each of which used input images of A549, HEK293, and NCI-H1975, respectively. The trained model was constructed in a web-based application so that it could be used in a web browser to provide an easily accessible user interface (https://bioanalysis-79545.web.app/main/images/a549). Based on the cell viability predicted from the microscopic cell image taken under a specific concentration of DOX treatment, IC50 was subsequently calculated using a custom algorithm that solved the Hill equation with four parameters. To obtain an accurate result of cell viability using the web-based application, the images should be captured using a sCMOS camera (Andor Technology) under × 100 magnification ratio.

### Prediction of cell viability using a CNN image analysis model

**Figure 2:**
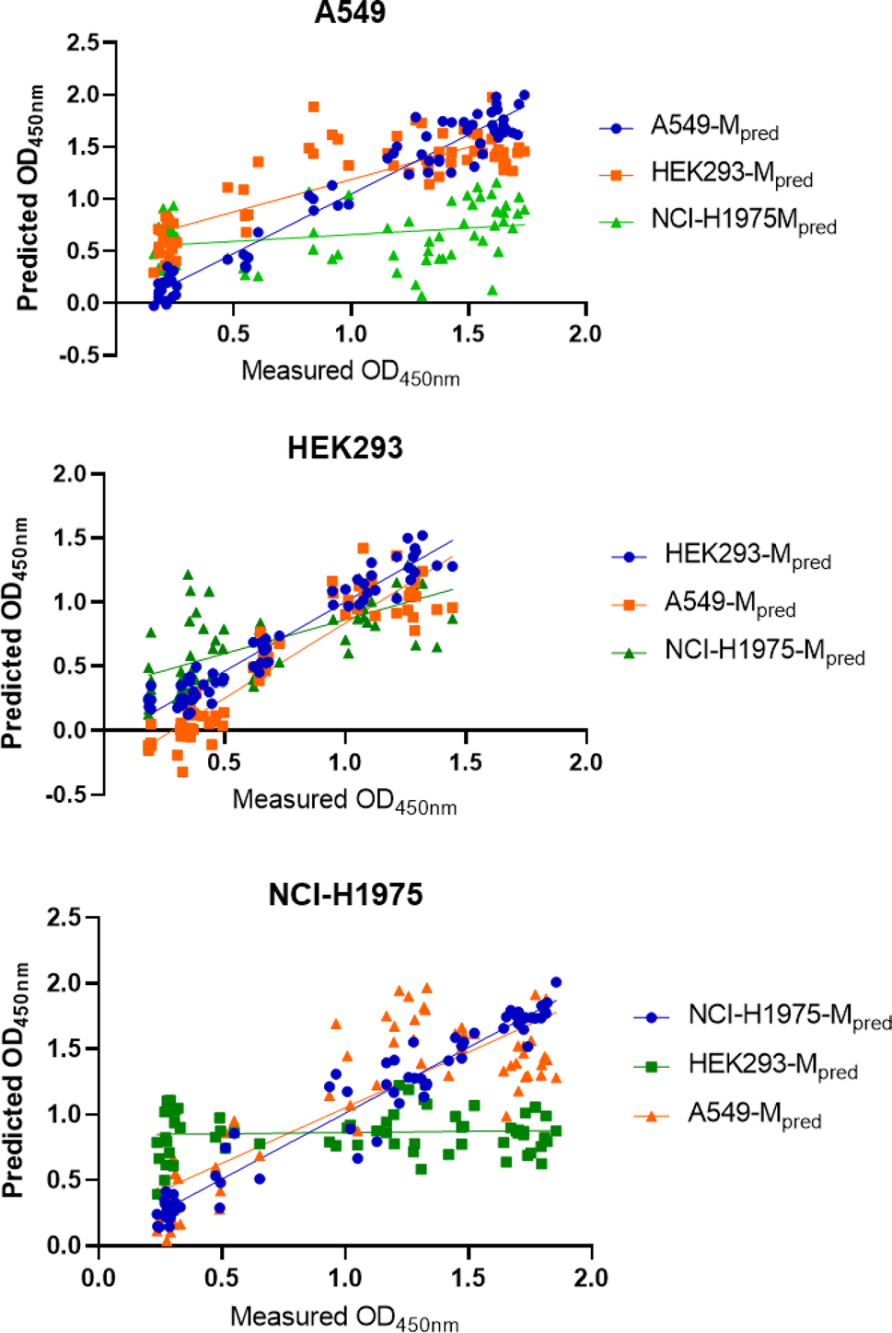
Correlation of measured and predicted optical density measured at 450 nm following colorimetric cell proliferation assay (CCK assay). The models constructed for the three types of cells were A549-M, HEK293-M, and NCI-H1975-M. The predicted values were drawn up from allogenic models as well as autologous models for cross validation. The linearity and statistical analysis results are shown in Table 1.

After training, the model was validated to determine whether the predictions based on label-free cell images were comparable to those of the conventional cell proliferation assay. The predicted values from the three types of models were plotted against the measured values obtained from the CCK assay. Linear regression analysis was performed to determine the correlation coefficient r^2^. Figure 2 shows the relationship between the measured and predicted values. The deviation from linearity was lowest when the OD_450 nm_ was estimated by the autologous model of each cell type; for example, the measured OD_450 nm_ values of A549 treated with various DOX concentrations were predicted more straightforwardly by A549-M, which was the model trained using A549 cell images. HEK293 and NCI-H1975 cells were also best fit by each autologous prediction model.

Table 1 shows the quantitative evaluation of the correlation between the measured and predicted values of OD_450 nm_ by statistical analysis. The correlation coefficient r^2^ values between the measured OD_450 nm_ of A549, HEK293, and NCI-H1975 and the values predicted by autologous models A549-M, HEK293-M, and NCI-H1975-M, were determined to be 0.9513, 0.9404, and 0.9491, respectively. These values are relatively higher than those estimated by allosteric prediction models: for example, decreased r^2^ (0.07962) was observed between measured OD_450 nm_ values for A549 cell viability and predicted OD_450 nm_ by the model trained using NCI-H1975 cell images (NCI-H1975-M). The slopes of the linear regression curves between the measured and estimated values using autologous prediction models were 1.138, 1.079, and 1.002 for A549, HEK293, and NCI-H1975, respectively. This result indicates that the relationship is almost comparable with the proportional ratio near ~ 1.0.

**Table 1:** Statistical analysis of linear relationship of measured and predicted OD_450 nm_.

Cell type	A549			HEK293			NCI-H1975		
Prediction model	A549-M	HEK293-M	NCI-H1975-M	HEK293-M	A549-M	NCI-H1975-M	NCI-H1975-M	A549-M	HEK293-M
Correlation, r^2^	0.9513	0.6996	0.07962	0.9404	0.8634	0.4474	0.9491	0.004085	0.7028
Equation	Y = 1.138*X − 0.09013	Y = 0.6197*X + 0.5682	Y = 0.1278*X + 0.5311	Y = 1.079*X − 0.07736	Y = 1.168*X − 0.3292	Y = 0.5299*X + 0.3333	Y = 1.002*X + 0.009382	Y = 0.01832*X + 0.8454	Y = 0.8457*X + 0.2078
Difference between measured and predicted values (Ave ± SD)	0.04874 ± 0.1714	0.1868 ± 0.3259	0.3435 ± 0.5708	0.02182 ± 0.1107	0.2119 ± 0.1930	0.004189 ± 0.2946	0.01163 ± 0.1370	0.04499 ± 0.3372	0.1904 ± 0.6037

The difference ± SD between the measured and predicted values was also determined, as shown in Table 1. The difference was calculated as 0.04874 ± 0.1714, 0.02182 ± 0.1107, and 0.01163 ± 0.1370 for A549, HEK293, and NCI-H1975, respectively, when each autologous model was used for estimation. The deviation of the prediction from the measured values increased over tenfold when the allosteric models were used to estimate cell viability. In the case of the difference between the measured and predicted values for HEK293 cell viability using the allogenic model of NCI-H1975, the average difference decreased to 0.0041, even though the autologous model prediction was 0.0218, which was attributed to the compensation effect of the summation of the difference in positive and negative values. The SD of the difference was still enhanced in the case of allogenic NCI-H1975-M.

**Figure 3:**
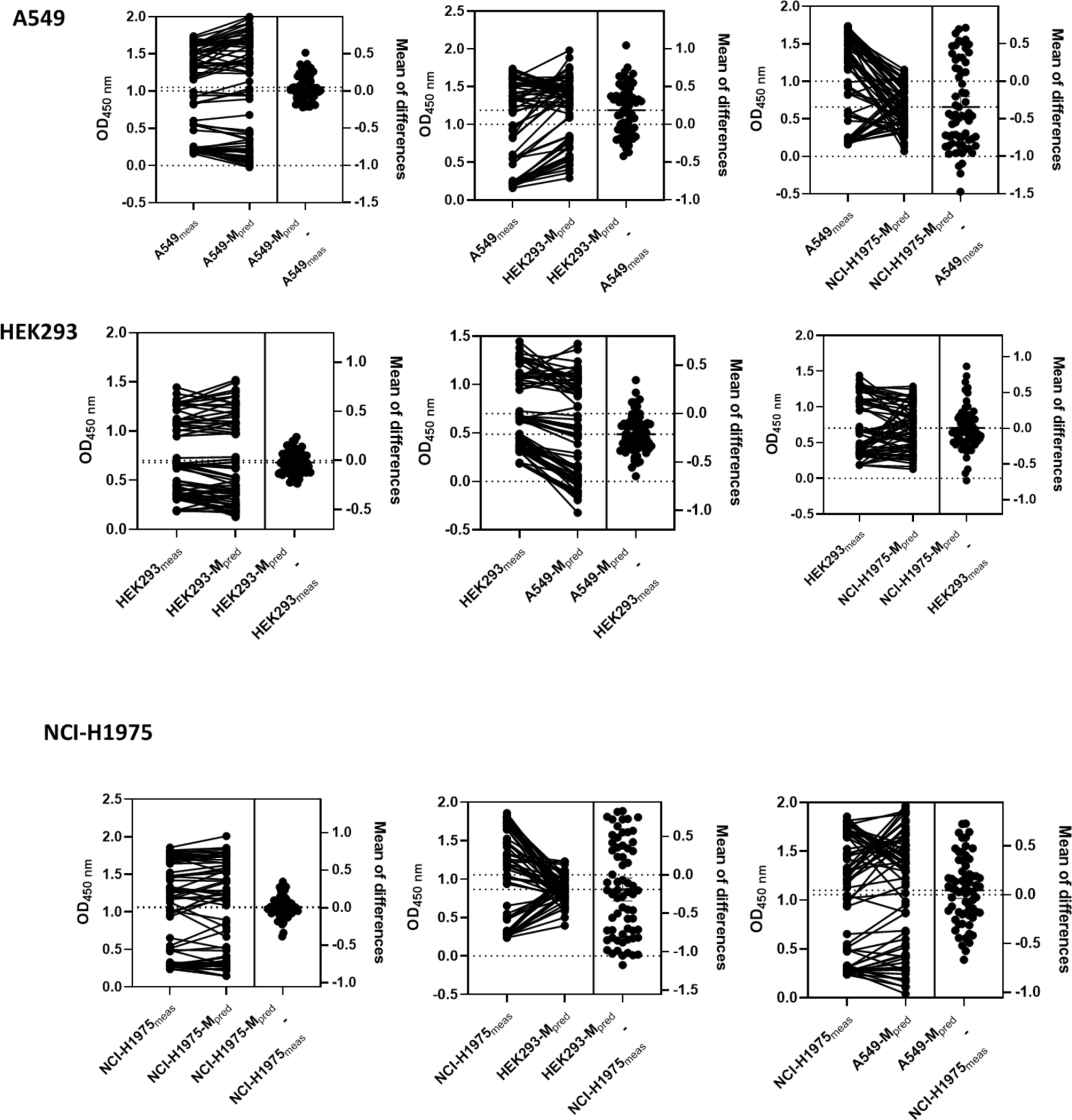
Correlation of measured and predicted optical density measured at 450 nm (OD_450 nm_) analyzed by paired *t* test. The corresponding measured and predicted values and difference of two values are plotted. The left panel for each cell type shows predicted values from autologous models; in the middle and right panels, these values are compared with the predicted values from allogenic models.

Figure 3 shows the distribution of the measured and predicted values based on the autologous and allogenic models. The difference between the measured and predicted values based on the autologous models showed a narrower distribution compared to that of the allogenic models, and the predicted values of OD_450 nm_ for A549, HEK293, and NCI-H1975 were more precisely matched to the estimated values from the respective autologous models A549-M, HEK293-M, and NCI-H1975-M.

In a previous study on the numerical deep learning of CNN to predict cell viability using label-free 3D cell images, the correlation coefficient r^2^ was determined to be 0.82–0.93^7^. In this case, cell images were assigned by fluorescence intensity following LIVE/DEAD cell staining of 1920 tumor sphere samples. In our study, the correlation coefficient r^2^ improved to over 0.94, and moreover, this was achieved by almost one-seventh of the training samples, more efficient than the previous study.

To support the improved accuracy even using the limited number of samples in this study, we additionally performed fourfold validation analysis by three types of CNN models, MobileNetV2, InceptionV3, and InceptionResNetV2. Table S1 shows the r^2^ coefficients between the measured and predicted values of OD_450 nm_ for A549 cells by fourfold cross validation. The results indicated that the r^2^ values were 0.9237, 0.9325, and 0.9331 for MobileNetV2, InceptionV3, and InceptionResNetV2 models, respectively, which were close to each other. Table S2 shows the results of fourfold cross validation using MobileNetV2 for A549, HEK293, and NCI-H1975 cells. The r^2^ values determined by the fourfold cross validation were 0.9237, 0.9218, and 0.9290 for A549, HEK293, and NCI-H1975, respectively, which were comparable to those determined by A549-M (0.9513), HEK293-M (0.9404), and NCI-H1975-M (0.9491), as shown in Table 1. These results supported the prediction power of the autologous models prepared in this study, which was achieved with total 264 samples for each cell line.

### Visualization of learned features

**Figure 4:**
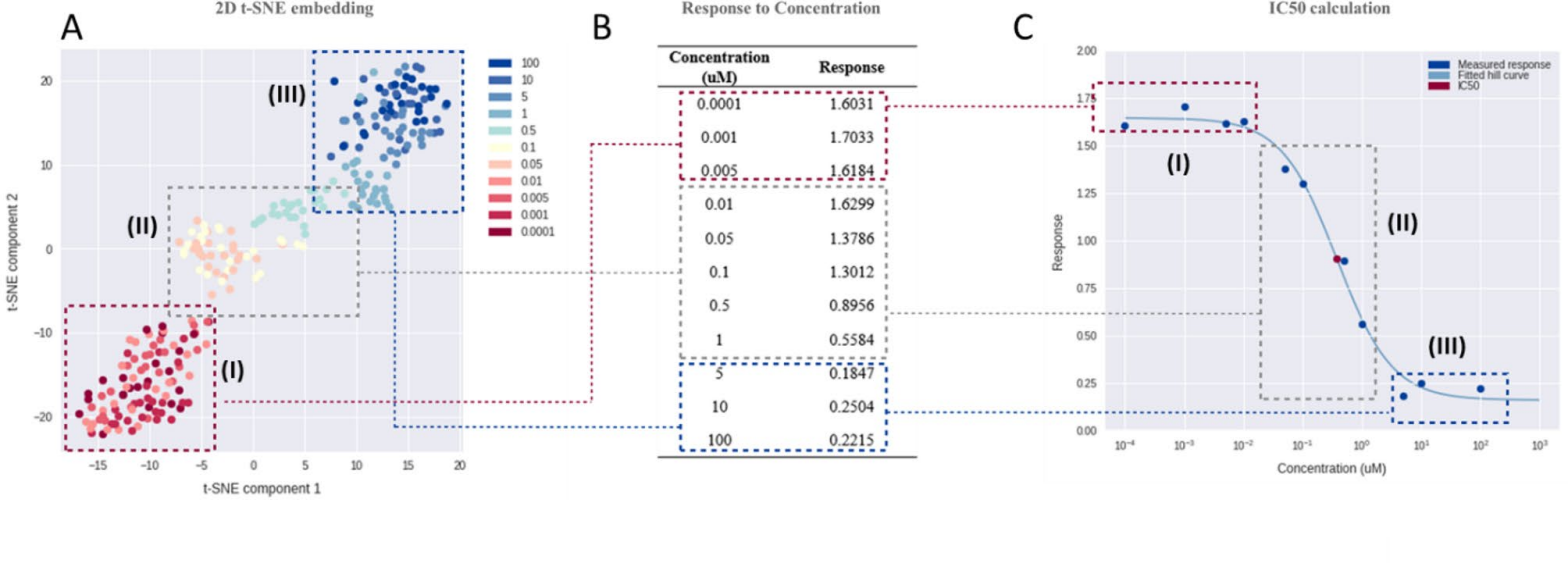
Feature visualization learned by MobileNetV2 for the entire dataset. (**A**) 1028-dimensional features of A549 cell images were projected to a 2D surface using t-SNE, and colored according to drug concentrations. (**B**) Responses to drug concentrations (OD_450 nm_). (**C**) Hill curve drawn using the concentrations and the response in (**B**). The image features of the top (I), slope (II), and bottom regions (III) of the Hill curve (**C**) are clustered in different sections of (**A**). The response values from the images in the slope region (II) increase according to the concentration, which is clearly reflected by the feature visualization (**A**).

To better investigate the classifiers discovered by deep learning, a visualization of the features linked to their biological meaning was presented, allowing for interpretation of the CNN model decisions. The extracted feature vectors of the test images in high-dimensional features by our trained model were transformed to lower-dimensional features by the well-known t-SNE^13^, as shown in Fig. 4A. Each dot corresponds to a test image, and the color is the treated DOX concentration. The plot of t-SNE shows the local structure of the high-dimensional input space. OD_450 nm_ measured by cell proliferation assay according to the DOX concentration in Fig. 4B represents the concentration-dependent drug responsiveness, which was fitted by a sigmoidal curve generated by the Hill equation (Fig. 4C). Based on the Hill curve in Fig. 4C, the IC50 concentration was determined for the concentration at which 50% cell viability was observed compared to that of control cells. Features from cell images corresponding to each concentration formed three clusters after transformation by t-SNE (I, II, and III in Fig. 4A), which were largely divided in the Hill curve into top (I), slope (II), and bottom (III) regions in Fig. 4C. The results implied that numerical values of cell viability by visual inspection of cell images could be learned, and that the trained model predicted cell viability in continuous numbers linked to cell images.

**Figure 5:**
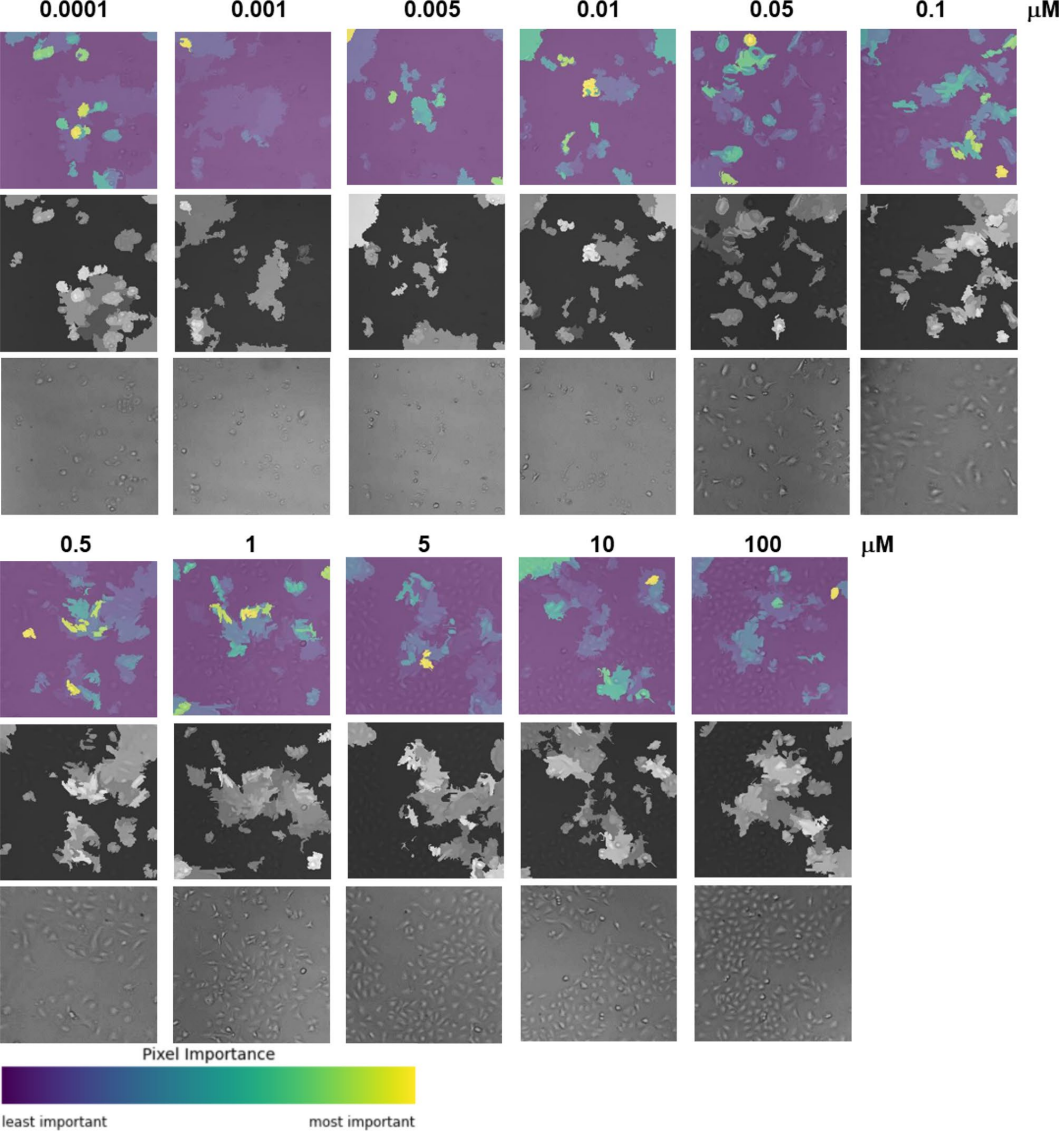
Heat map of important features in the input cell images for the decision of cell viability generated using the prediction model A549-M.

Another visualization technique is to reveal the localization of discriminative regions for decisions in an input image. Google explainable AI was utilized to understand how our trained model predicted drug responsiveness from cell images. The XRAI option of explainable AI was used to display attributions, which was highlighted in accordance with the prominent image features that were impactful in the model rather than the individual pixels. XRAI highlights the most influential regions in yellow and the least influential in blue, based on the viridian color palette, as shown in Fig. 5. The localization of the attributions in the input images shows that the prominent features of the decision overlapped with the area of viable cells. This result complemented the t-SNE visualization by localizing important features in the final convolutional layer of the CNN.

### Prediction of IC50 using label-free cell images

To calculate the IC50, images corresponding to various drug concentrations were required as input data. We used 11 drug concentrations to treat three types of cells, and captured the images of cells in each well. The histogram of the response values (assigned by cell viability) for the 198 training data samples are shown in Fig. S3. The responses were not uniformly distributed but covered a sufficiently large area.

**Figure 6:**
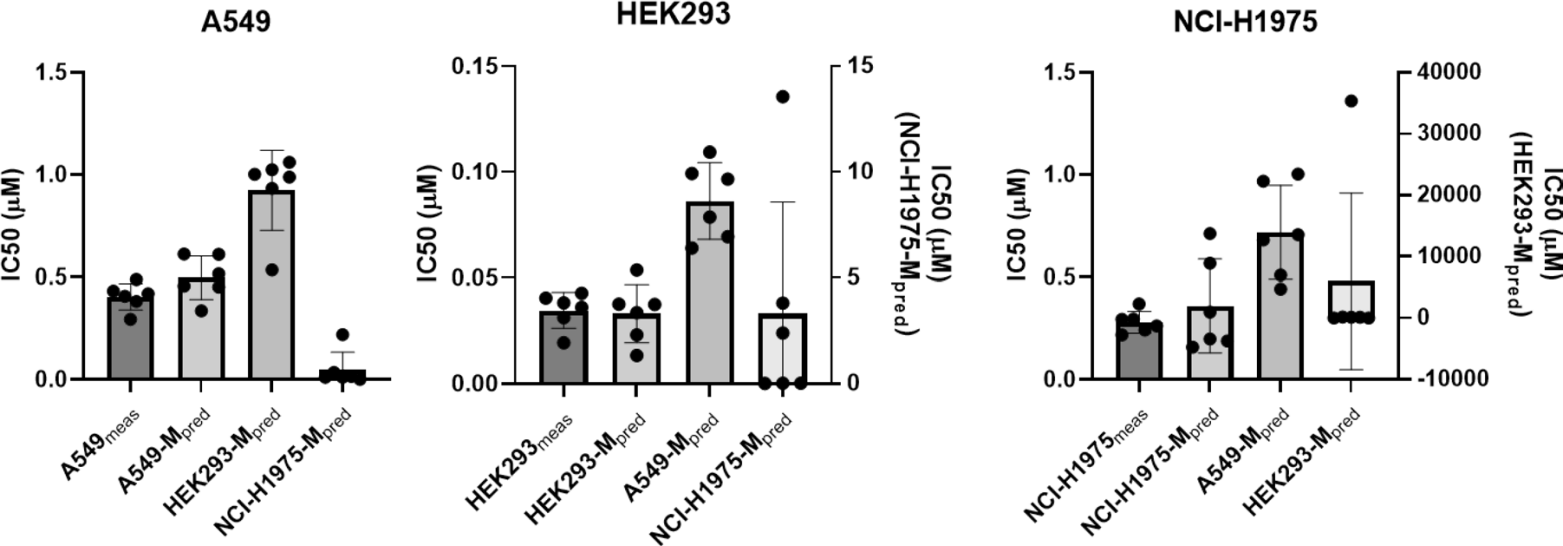
Distribution of IC50 values from 6-repeated tests of doxorubicin effects. Measured and predicted IC50 for three cell types compared to each other. The results of the statistical analysis are provided in Table 2.

Figure 6 shows the measured and predicted IC50 values, which were estimated using autologous and allogenic prediction models. For the three cell types, IC50_pred_ estimated by the autologous model was closest to IC50_meas_ for each cell line. The accuracy of IC50_pred_ reflected the precise cell viability estimated by the CNN models. This result indicated that prediction of drug responsiveness as IC50 was available, using the method of label-free imaging analysis through the numerical deep learning developed in this study.

Table 2 shows the IC50 concentration for DOX exposure obtained from the CCK assay (IC50_meas_) and prediction by CNN models (IC50_pred_) for A549, HEK293, and NCI-H1975 cells. The average difference between IC50_meas_ and IC50_pred_ for the autologous model was 0.0935, 0.0015, and 0.0785 for A549, HEK293, and NCI-H1975 cells, respectively. However, when the estimation was performed using the allogenic training model, the difference between IC50_meas_ and IC50_pred_ increased from several to hundreds of times over. In addition, unpaired *t* tests showed that IC50_meas_ and IC50_pred_ were not significantly different if autologous models were used to estimate IC50_pred_ (*p* = 0.094, 0.8205, 0.4334 for A549, HEK293, and NCI-H1975, respectively). In other cases, such as IC50_pred_ of A549 cells using HEK293-M and NCI-H1975-M, the unpaired *t* test showed that there was a significant difference compared to IC50_meas_ (*p* = 0.001 and p < 0.0001, respectively). For HEK293, IC50_meas_, and IC50_pred_ estimated from allogenic NCI-H1975-M showed no statistically significant difference (*p* = 0.1597). This result was attributed to the estimated values of HEK293 IC50_pred_ using NCI-H1975-M showing wide variation: the difference between IC50_meas_ and IC50_pred_ was 3.266 ± 5.266, which was almost a hundred times higher than that of IC50_pred_ using the autologous model.

**Table 2:** Averages and differences between IC50_meas_ and IC50_pred_ for doxorubicin effects (n = 6).

Cell type	A549			HEK293			NCI-H1975		
Prediction model	A549-M	HEK293-M	NCI-H1975-M	HEK293-M	A549-M	NCI-H1975-M	NCI-H1975-M	A549-M	HEK293-M
IC50_meas_	0.4035 ± 0.0642			0.0346 ± 0.0085			0.2801 ± 0.0529		
IC50_pred_	**0.4970 ± 0.1062**	0.9236 ± 0.1956	0.0484 ± 0.0846	**0.0331 ± 0.0138**	0.0861 ± 0.0181	3.3010 ± 5.2675	**0.3586 ± 0.2296**	0.7183 ± 0.2302	5977 ± 14,392
Difference between measured and predicted values (Ave ± SD)	**0.0935 ± 0.0602**	0.5201 ± 0.2015	− 0.3551 ± 0.06613	− **0.0015 ± 0.0126**	0.0515 ± 0.0184	3.266 ± 5.266	**0.0785 ± 0.2429**	0.4382 ± 0.2425	5977 ± 14,392
*p*-value (unpaired *t* test)	**0.0947**	0.0001	< 0.0001	**0.8205**	< 0.0001	0.1597	**0.4334**	0.0011	0.3330
*p*-value (paired *t* test)	**0.0126**	0.0015	< 0.0001	**0.7765**	0.0010	0.1892	**0.4645**	0.0069	0.3557

Prediction values from autologous models are in bold.

In the paired *t* test, the estimated values of IC50_pred_ of DOX for HEK293 (*p* = 0.7765) and NCI-H1975 (*p* = 0.4645) from autologous models were not significantly different compared to IC50_meas_ values of HEK293 and NCI-H1975 cells, respectively. For A549 cells, p-values for measured and autologous model prediction of IC50 were different in the paired *t* test (*p* = 0.012); however, the difference of IC50_pred_ to IC50_meas_ for A549 from allogenic models, HEK293-M and NCI-H1975-M, was dramatically enhanced, which was reflected by statistical parameter, *p* = 0.0015 and < 0.0001, respectively.

As a result, the predicted IC50 values for DOX exposure were comparable to that of the IC50 values measured by colorimetric assay, because there was no statistical difference between the distribution of IC50_meas_ and IC50_pred_ by the paired and unpaired *t* test (the only exception was the paired *t* test for A549 cells). The results indicated that the CNN model predicted the cell viability sufficiently precisely to determine drug responsiveness statistically, but the prediction capability could degrade when the numerical learning was performed using cell images from different types of cells.

Table S3 shows IC50_pred_ of A549 cells determined by three CNN models (MobileNetV2, InceptionV3 and InceptionResNetV2) by fourfold cross validation. When IC50_meas_ was 0.3747, IC50_pred_ values were determined as 0.3939, 0.3753, and 0.3163, and the average difference (IC50_pred_ − IC50_meas_) was 0.01928, 0.00067 and − 0.0535, by MobileNetV2, InceptionV3 and InceptionResNetV2, respectively. In this case, three types of CNN models estimated IC50_pred_ similar to IC50_meas_, without statistical difference (*p* > 0.05). Table S4 shows the IC50_pred_ for A549, HEK293, NCI-H1975, determined by fourfold cross validation using MobileNetV2. The average difference between IC50_meas_ and IC50_pred_ (IC50_pred_ − IC50_meas_) for A549, HEK293, NCI-H1979 were 0.01928, 0.00202, and 0.03274, respectively. The paired and unpaired *t* test indicated that the distribution of IC50_meas_ and IC50_pred_ values was not statistically different (*p* > 0.05). These results supported the IC50 prediction capability using A549-M, HEK293-M, and NCI-H1975-M provided in Table 2.

There are several reports to predict IC50 of anti-cancer drugs using genomic profiles and drug fingerprints^18,19^. In these studies, genomic profiles of various cancer cell lines and fingerprints of drugs were trained to categorize IC50 levels in three steps, high responsiveness (class 0), intermediate responsiveness (class 1), and low responsiveness (class 2). The genomic profiles could be an excellent input data to predict IC50 values, because response to drugs in cancers are known to be closely related to genetic variations by pharmacogenomic mechanisms. However, the genomic profiles require time and cost to obtain, compared to the captured images of cells following an exposure to drugs. The present models developed in this study involved simple, cheap, and prompt methods to prepare input datasets, and furthermore, could be exploited in well-established assay methods to determine cell viability and IC50.

## Conclusion

We constructed cell viability-based IC50 prediction models for three cell types: A549, HEK293, and NCI-H1975. These cells originated from different tissues and diseases, which have different morphologies and IC50 values. In this context, three models were trained with different input images, and the prediction capabilities were cross-validated to determine the specificity of the models according to the input cell images. The results showed that the model accuracy was dependent on the input data: by using autologous input data with the corresponding model, more accurate prediction results were achieved (r^2^ > 0.94).

Recently, a CNN model was shown to predict drug responsiveness in IC50 by examining 3D tumor spheroids to determine responsivity of anticancer drugs such as doxorubicin, oxaliplatin, and irinotecan^7^. The ground truth value of the cell viability was determined by fluorescence intensity followed by LIVE/DEAD cell staining, which was used as the input image layer. In another study, fluorescent LIVE/DEAD cell classification was performed in image processing, using deep learning and taking bright-field images as input^5^. The ground truth label for each cell was set by LIVE/DEAD cell fluorescence staining and paired to the bright-field images, which were used to train the prediction model. In this case, the quantitative assessment of cell growth by drug treatment was not exploited. Until now, non-labeled microscopic images and numerical values determined by conventional cell proliferation assays has not been applied to determine IC50 values by deep-learning approaches. In this study, numerical deep learning models constructed with those input datasets could be proposed as an alternative method to replace colorimetric cell proliferation assays.

In this study, we used over 200 image data samples, which is a relatively low number of images for deep learning. However, the augmentation of data during pre-processing was effective in improving the prediction accuracy of the models. In addition, the visualization of important image features for model decisions supported the classification results by t-SNE and the heat map of the images.

Our model does not require any labeling of probes; thus, using cell images from bright field microscopy of cell culture dishes, drug responsiveness could be determined rapidly and with low cost and labor. The cultured cells used for the estimation of drug responsiveness are still available for further study.

## Supplementary Information

Supplementary Information.
