# Supplementary material for: Numerical learning of deep features from drug-exposed cell images to calculate IC50 without staining
Source: Sci Rep. 2022 Apr 22;12:6610. doi: 10.1038/s41598-022-10643-9 (PMC9033873; doi:10.1038/s41598-022-10643-9)
Supplement: Supplementary file 1 — Supplementary Information. [file 41598_2022_10643_MOESM1_ESM.docx]

**Supplementary Information**

Numerical learning of deep features from drug-exposed cell images to calculate IC50 without staining

Koorae Cho,^†^ Eun-Sook Choi,^†^ Jung-Hee Kim, Jong-Wuk Son,^*^ Eunjoo Kim^*^

*Division of Electronics and Information System Research, Daegu Gyeongbuk Institute of Science and Technology (DGIST), Techno-Jungangdaero 333, Daegu, Republic of Korea, 42988*

^†^ These authors contributed equally to this work.

Correspondence to:

Eunjoo Kim (ejkim@dgist.ac.kr)

Division of Electronics and Information System Research, Daegu Gyeongbuk Institute of Science and Technology, Techno-Jungangdaero 333, Daegu, Republic of Korea, 42988

+82-53-785-2530

Jong-Wuk Son (jwson@dgist.ac.kr)

Division of Electronics and Information System Research, Daegu Gyeongbuk Institute of Science and Technology, Techno-Jungangdaero 333, Daegu, Republic of Korea, 42988

+82-53-785-4520

Table S1. Correlation of measured and predicted OD_450nm_ for A549 cells determined by 4-fold cross validation using three different CNN models.

| Cell type |  | A549 | | | | |
| --- | --- | --- | --- | --- | --- | --- |
| CNN Model |  | MobileNetV2 |  | InceptionV3 |  | Inception-ResNetV2 |
| Correlation, r^2^ |  | 0.9237 |  | 0.9325 |  | 0.9331 |
| Equation |  | Y = 0.9638*X + 0.08939 |  | Y = 0.9470*X - 0.00065 |  | Y = 0.9182*X + 0.02762 |
| Difference between measured and predicted values  (Ave± SD) |  | 0.05213 ± 0.1690 |  | -0.04795 ± 0.1575 |  | -0.05653 ± 0.1568 |

Table S2. Correlation of measured and predicted OD_450nm_ for three types of cells by 4-fold cross validation using MobileNetV2 model.

| CNN Model |  | MobileNetV2 | | | | |
| --- | --- | --- | --- | --- | --- | --- |
| Cell type |  | A549 |  | HEK293 |  | NCI-H1975 |
| Correlation, r^2^ |  | 0.9237 |  | 0.9218 |  | 0.9290 |
| Equation |  | Y = 0.9638*X + 0.08939 |  | Y = 0.9882*X  - 0.05068 |  | Y = 1.069*X  - 0.08374 |
| Difference between measured and predicted values  (Ave± SD) |  | 0.05213 ± 0.1690 |  | 0.04223 ± 0.1142 |  | -0.01064 ± 0.1765 |

Table S3. Correlation of IC50_meas_ and IC50_pred_ of A549 cells for doxorubicin effects using three different CNN models (n=24, 4-fold cross validation).

| Cell type |  | A549 | | | | |
| --- | --- | --- | --- | --- | --- | --- |
| CNN Model |  | MobileNetV2 |  | InceptionV3 |  | Inception-ResNetV2 |
| IC50_meas_ |  | 0.3747 | | | | |
| IC50_pred_ |  | 0.3939 |  | 0.3753 |  | 0.3163 |
| Difference between measured and predicted values  (Ave± SD) |  | 0.01928  ±0.042 |  | 0.0006758  ±0.04105 |  | -0.05835  ±0.037 |

| *p*-value  (unpaired t-test) |  | 0.6545 |  | 0.9869 |  | 0.1291 |
| --- | --- | --- | --- | --- | --- | --- |

| *p*-value  (paired t-test) |  | 0.6025 |  | 0.9838 |  | 0.0709 |
| --- | --- | --- | --- | --- | --- | --- |

Table S4. Correlation of IC50_meas_ and IC50_pred_ of three types of cells for doxorubicin effects using MobileNetV model (n=24, 4-fold cross validation).

| CNN Model |  | MobileNetV | | | | |
| --- | --- | --- | --- | --- | --- | --- |
| Cell type |  | A549 |  | HEK293 |  | NCI-H1975 |
| IC50_meas_ |  | 0.3747 |  | 0.03354 |  | 0.3048 |
| IC50_pred_ |  | 0.3939 |  | 0.03557 |  | 0.3375 |
| Difference between measured and predicted values  (Ave± SD) |  | 0.01928  ±0.042 |  | 0.002028  ±0.004864 |  | 0.03274  ±0.030 |
| *p*-value  (unpaired t-test) |  | 0.6545 |  | 0.6787 |  | 0.2889 |
| *p*-value  (paired t-test) |  | 0.6025 |  | 0.6197 |  | 0.2353 |


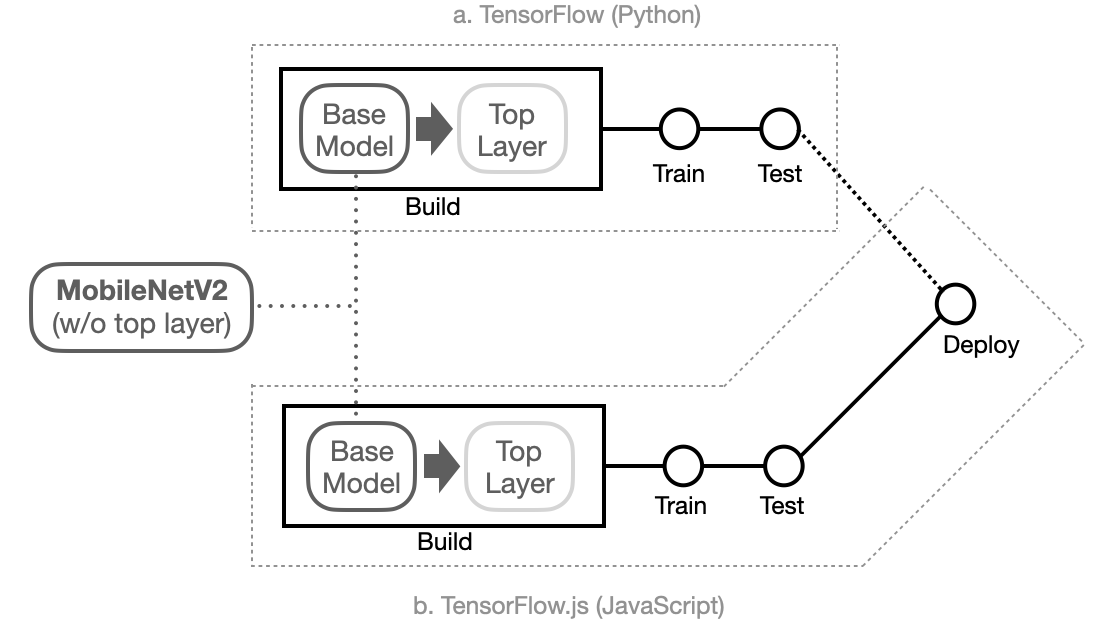


Figure S1. Model building, training and deploying. Model is built and trained in TensorFlow and converted to TensorFlow.js format.


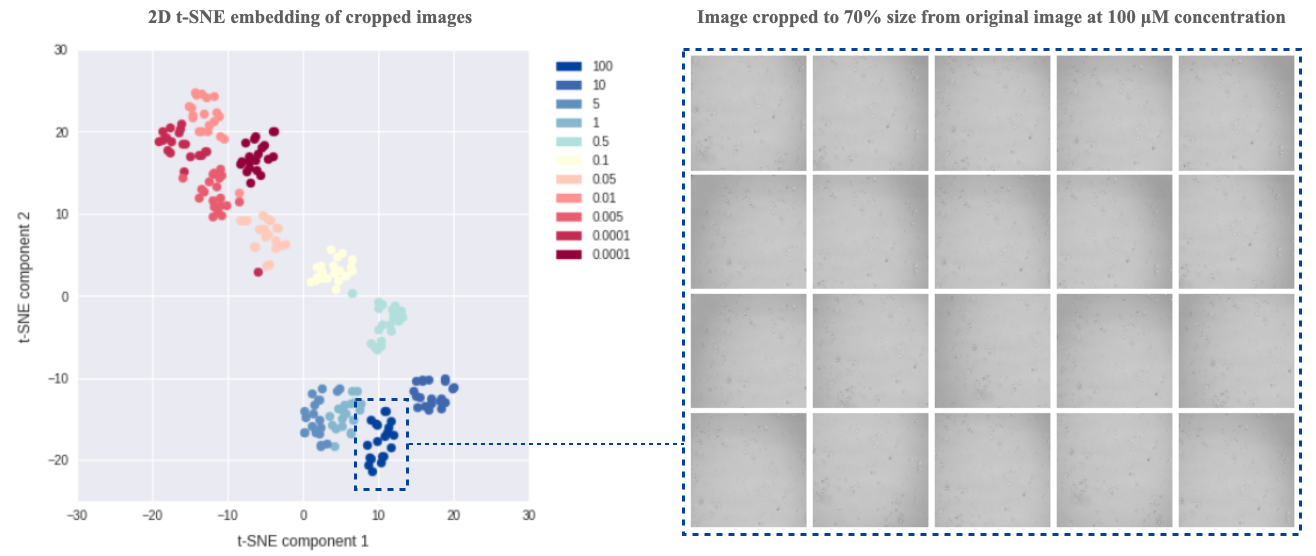


Figure S2. Feature visualization learned by MobileNetV2 for augmented images of A549 cell image, colored according to drug concentrations. Twenty images were randomly cropped from original image at 70% size. Images augmented at the same concentration still form clustering, and there is a distance between different clusters.


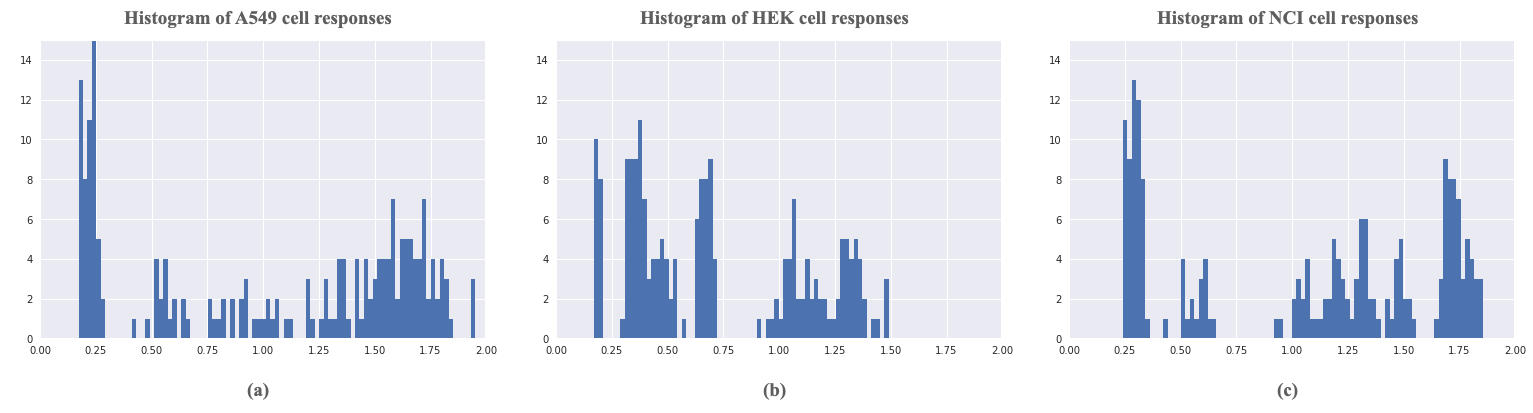


Figure S3. Histograms of cell responses for 198 training data (labels). Bin size for histogram are set to 0.02.
